# Robotic versus open Frey’s procedure for chronic pancreatitis: a single center’s experience of 40 cases over a 7-year period

**DOI:** 10.1186/s12876-026-04790-w

**Published:** 2026-04-17

**Authors:** Wei Li, Yali Cheng, Xue Yang, Qingyong Ma, Zheng Wu, Zheng Wang

**Affiliations:** Department of Hepatobiliary Surgery, The First Affiliated Hospital of Xi’an Jiaotong University, 277 West Yanta Road, Xi’an, Shaanxi 710061 P.R. China

**Keywords:** Chronic pancreatitis, Frey’s procedure, Robotic procedure, ESWL, Surgery

## Abstract

**Background:**

Chronic pancreatitis (CP) is a progressive inflammatory disease characterized by structural damage and chronic pancreatic fibrosis. Frey’s procedure combined both resection and drainage, is a safe and effective procedure for chronic pancreatitis.

**Methods:**

This study aims to compare the effect of robotic and open Frey’s procedure in our single center. A retrospective study of patients who underwent a Frey’s procedure (robotic and open) due to CP between January 2016 to December 2022 in a hospital in Xi’an Jiaotong University was made.

**Results:**

In our study, 40 patients met the inclusion criteria, 72.5% of patients were male. The etiology in most cases (47.5%) was idiopathic, and 32.5% of patients were alcoholic. 22.5% patients received extracorporeal shock wave lithotripsy (ESWL) before surgery. 21/40 patients underwent robot assisted Frey’s procedure. The duration of surgery was longer in robotic group (315 min vs. 238 min, *p* = 0.004). The median length of in-hospital stay and postoperative hospital stay were shorter in the robotic group. There was no significant difference in the postoperative complications and short-term outcomes of pancreatic exocrine and endocrine dysfunction between the two groups. The rate of pain relief was 100%. Although no significant difference was observed in the operation time and postoperative complications between the patient who got pre-operative ESWL or not, patients who received ESWL before surgery were most likely to have easily stones removed.

**Conclusions:**

Robotic Frey’s procedure is safe and feasible for CP patients. Preoperative ESWL treatment may be helpful for intraoperative pancreatic duct stone removal.

## Introduction

 Chronic pancreatitis (CP) is a progressive inflammatory disease that leads to structural damage and fibrosis that can eventually result in exocrine and endocrine dysfunction of the pancreas [1]. The disease can negatively affect quality of life and is reported to have an annual incidence of 4–14 cases per 100,000 people worldwide [2].

Intractable pain is the most distressing symptom of CP [3]. Therefore, the primary goal of management of CP is control of pain. There is still debate about the best treatment for CP. Current literature recommends conservative measures for initial treatment, such as analgesia, dietary changes, pancreatic supplementation and smoking cessation [4]. This approach is largely based on the paradigm that CP is a self-limiting disease [5]. However, up to 50% of patients may receive surgical intervention at some point during their disease [6]. Current evidence suggests that surgery is more effective than endoscopic therapy in terms of more rapid, effective and sustained pain relief. In addition, several recent studies have shown that early surgical intervention can provide better long-term pain control and preserve exocrine and endocrine function [7, 8].

Frey’s procedure is a duodenum preserving head resection combined with lateral pancreaticojejunostomy [9]. It is one of the most commonly performed procedures for chronic pancreatitis, which can be performed open, laparoscopic and also feasible robotically. This technique has reasonable pain control during long-term follow-up and is associated with a better quality of life, shorter surgical time and a lower incidence of postoperative complications compared to other CP procedures [10]. However, data on robot assisted Frey’s procedure is scarce.

The primary aim of this study was to report the surgical experience on CP of 40 patients who underwent Frey’s procedure (robotic and open) at the first affiliated hospital of Xi’an Jiaotong University in China between 2016 and 2022. Secondary aims were to compare the clinical characteristics of robotic and open Frey’s procedure in our single center and if pre-operative extracorporeal shock wave lithotripsy (ESWL) will affect the whole procedure or not.

### Patients and methods

This is a retrospective case series study conducted at the First Affiliated Hospital of Xi’an Jiaotong University in Xi’an of China. Patients who underwent Frey’s procedure (robotic or open) due to CP at the First Affiliated Hospital of Xi ‘an Jiaotong University between January 2016 to December 2022 were included in the study.

The study protocol was approved by the Ethics Committee of the First Affiliated Hospital of Xi’an Jiaotong University and the study was conducted in accordance with the approved guidelines. Our study complied with the relevant requirements of the Declaration of Helsinki. Informed consent was obtained from all subjects and/or their legal guardian(s).

Demographic, clinical, radiological, and follow-up data were obtained from a prospectively maintained database, with additional retrospective review. For each patient, the diagnosis was confirmed by an experienced pathologist. Medical records of these patients were reviewed for data that include demographics, etiology, ASA scores, pre-operative serum analysis, clinical symptoms, pre-operative ESWL, operative time, postoperative complications and so on.

Data were expressed as mean ± standard deviation (SD) and n refers to the number of patients. Unpaired Student’s t-test was applied to compare two sets of data. One-way analysis of variance (ANOVA) with Dunnett’s post-test was used for multiple comparisons. Data were statistically analyzed using SPSS version 22.0 software (IBM Inc.; Armonk, NY, USA). *P* < 0.05 was set as statistical significance. Group differences with continuous data were analyzed using independent samples t test.

## Results

### Clinical characteristics of patients with CP

The final study population included 40 CP patients who underwent surgery for robotic or open Frey’s procedure. Among them, 29 were male (72.5%) and 11 were female (27.5%), with a median age at diagnosis of 41.3 years (Table 1). The most common etiologies were alcoholic (32.5%), recurrent acute pancreatitis (20%)and idiopathic (47.5%). The clinical symptoms at admission and the frequency are presented in Table 1. Abdominal pain (82.5%), Hyperglycemia (10%) are two most shown symptoms (Table 1). Three patients (7.5%) were asymptomatic. ASA I/II scores accounted for the majority of 87.5%. The remaining 12.5% is classified as ASA III/IV scores (Table 1).

**Table 1:** Demographics of 40 chronic pancreatitis patients

Variable	Value
Age (year)	41.3
Male Gender	72.5%
Body Mass Index	21.5
Etiology	
Alcoholic	32.5%
Recurrent acute pancreatitis	20%
Idiopathic	47.5%
Symptom	
Abdominal pain	82.5%
Hyperglycemia	10%
No symptoms	7.5%
Duration of symptoms (month)	32
ASA Scores	
I / II	87.5%
III / IV	12.5%
Diabetes Mellitus	35%
Steatorrhea	12.5%
Pre-operative serum analysis	6.2
Hemoglobin (g/L)	131.7
White blood cell count	6.24
Albumin (g/L)	39.7
CA 19 − 9 (U/mL)	14.1
Pre-operative ESWL	22.5%

*ESWL* Extracorporeal shock wave lithotripsy

**Fig. 1:**
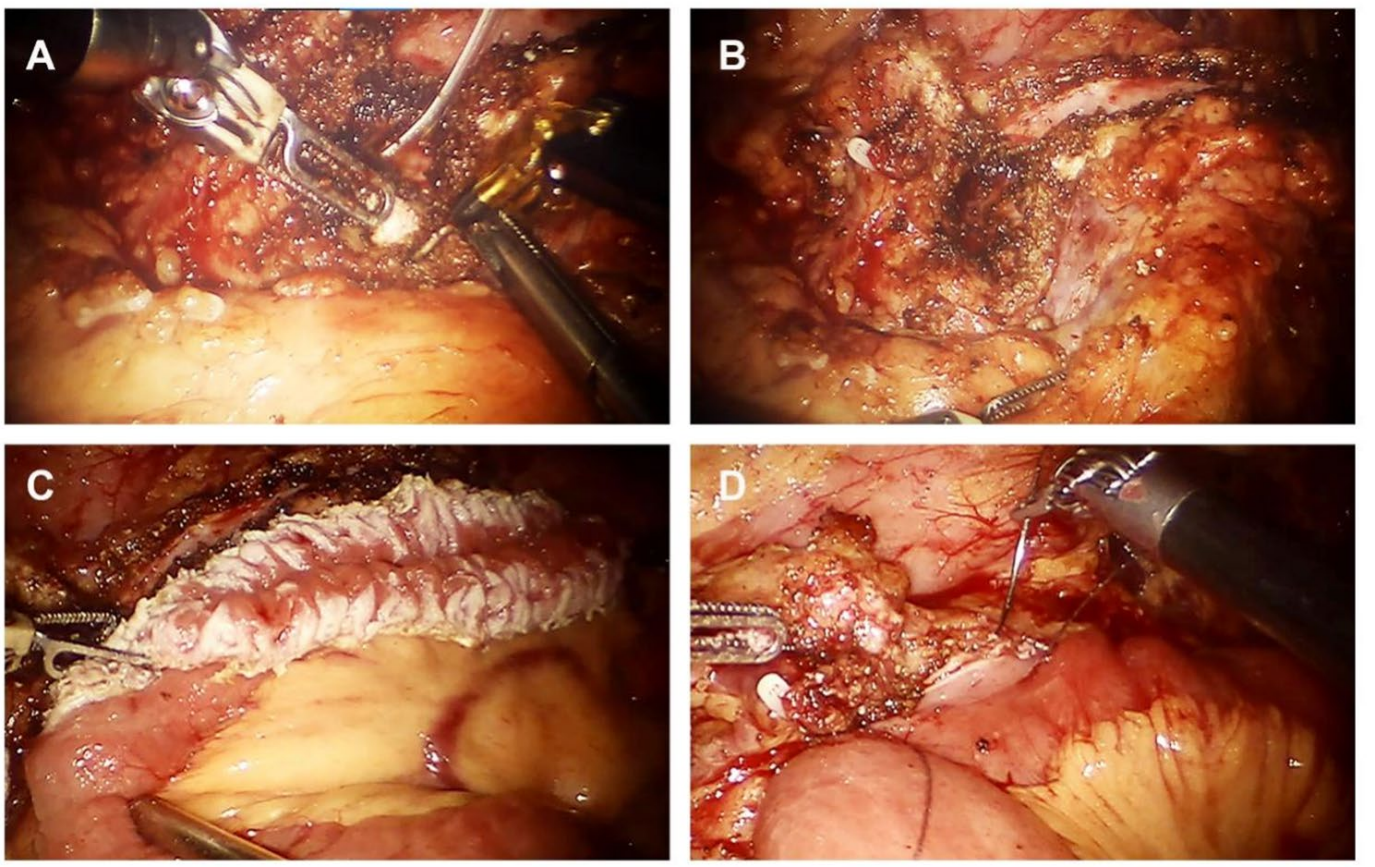
The robot-assisted Frey’s surgery procedure. **A** Opening of main pancreatic duct and retrieving the stone; (**B**) A longitudinal opening of pancreas from the head to the body; (**C**) A longitudinal opening of the jejunum; (**D**) Side-to-side pancreaticojejunostomy

**Fig. 2:**
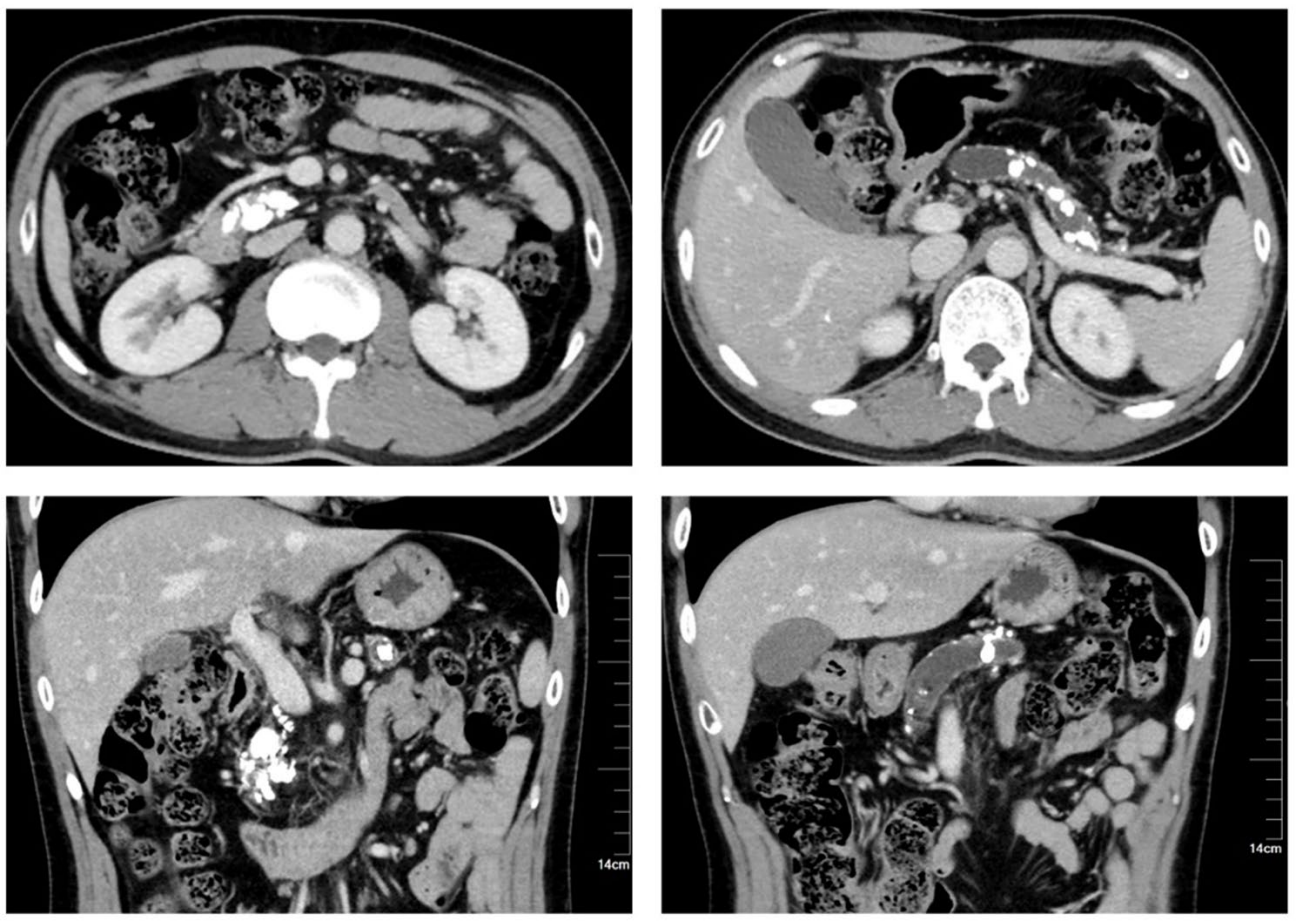
Computed tomography scan of the abdomen showing multiple pancreatic stone with pancreatic ductal dilatation and parenchymal atrophy of the pancreas

Before the operations, 35% of CP patients had Diabetes Mellitus and 12.5% of patients had steatorrhea. In addition, 22.5% of patients received pre-operative ESWL. Several biochemical tests were performed before the operation. The hemoglobin level was 131.7 g/L. The white blood cell count was 6.24 10^9^/L. The albumin level was 39.7 g/L and the CA 19 − 9 level was 14.1 U/mL. The procedure of robot-assisted Frey’s procedure was shown in Fig. 1. Computed tomography images show multiple pancreatic stone with pancreatic ductal dilatation and parenchymal atrophy of the pancreas (Fig. 2).

### The pre-operative characteristics of CP patients who underwent robot-assisted and open Frey’s procedures

Among the 40 CP patients, there were no significant difference in age, gender, etiology, symptoms and other indicators between the robot-assisted Frey’s procedure group and the open Frey’s procedure group (Table 2). In terms of etiology, both groups were mainly idiopathic, followed by alcoholic and recurrent acute pancreatitis. Before the surgery, there was no significant difference in symptoms between the robotic and open Frey’s surgery groups. The same situation occurred in ASA scores. ASA I/II scores accounted for the majority in both groups, while ASA III/IV scores were only a minority. Pre-operative serum analysis, such as hemoglobin, white blood cell count, albumin and CA 19 − 9 levels also did not differ significantly between the robot-assisted and open Frey’s procedure groups. Seven of the robot-assisted Frey’s procedure patients underwent ESWL before surgery. While only 2 of the open Frey’s procedure patients received ESWL before surgery (Table 2).

**Table 2:** Demographic and pre-operative characteristics of patients who underwent robot-assisted and open Frey’s procedure

Variable	Robot-assisted Frey’s procedure (*n* = 21)	Open Frey’s procedure (*n* = 19)	*p* value
Age (year)	38.3 (15–63)	44.5 (14–69)	0.182
Male Gender	66.7% (14)	78.9% (15)	0.385
Body Mass Index	21.3	21.7	0.704
Etiology			0.767
Alcoholic	6	7	
Recurrent acute pancreatitis	5	3	
Idiopathic	10	9	
Symptom			0.531
Abdominal pain	16	17	
Hyperglycemia	3	1	
No symptoms	2	1	
Duration of symptoms (month)	33.4 (1–120)	30.5 (0.5–144)	0.827
ASA Scores			0.654
I / II	19	16	
III / IV	2	3	
Diabetes Mellitus	9 (42.9%)	5 (26.3)	0.273
Steatorrhea	4 (19.0%)	1 (5.3%)	0.345
Pre-operative serum analysis			
Hemoglobin (g/L)	135.1	127.9	0.248
White blood cell count	6.6	5.9	0.428
Albumin (g/L)	40.5	38.7	0.267
CA 19 − 9 (U/mL)	11.2	17.2	0.077
Pre-operative ESWL	7 (33.3%)	2 (10.5%)	0.133

*ESWL* Extracorporeal shock wave lithotripsy

### The operative characteristics of CP patients who underwent robot-assisted and open Frey’s procedures

The most significant difference between the CP patients receiving robot-assisted or open Frey’s procedures was operative time, in-hospital stay and postoperative hospital stay (Table 3). The time required for robot-assisted Frey’s surgery (315 min) was significantly longer than that for open Frey’s surgery (238 min, *P* = 0.004). The in-hospital stays (14.4 days) and postoperative hospital stays (8.1 days) for robot-assisted Frey’s procedures were also shorter than those for open Frey’s procedures (18.9 days, *P* = 0.001 and 11.1 days, *P* = 0.002). Other intraoperative symptoms such as intraoperative bleeding, intraoperative transfusion and pseudocyst drainage were almost the same in both groups. Postoperative complications such as postoperative pancreatic fistula, surgical site infection, delayed gastric emptying and upper gastrointestinal bleeding were basically the same between the two groups. However, there was more trend of the complications in the open Frey’s group than robot-assisted Frey’s group but without statistical significance (Table 3). The reintervention rate and mortality rate were 0 in both groups after surgery. There was 1 case each in the two groups that developed post-operative exocrine pancreatic dysfunction. The pain of both groups of patients had been relieved during the post-operative period. No one reported any symptoms of abdominal pain or steatorrhea on follow-up after 6 months. Neither group required insulin replacement.

**Table 3:** Operative characteristics and early outcomes of patients who underwent robot-assisted and open Frey’s procedures

Variable	Robot-assisted Frey’s procedure (*n* = 21)	Open Frey’s procedure (*n* = 19)	*p* value
Operative time (min)	315 (180–540)	238 (150–360)	0.004
Intraoperative bleeding (mL)	147 (50–600)	189 (50–600)	0.351
Intraoperative transfusion	0 (0%)	2 (10.5%)	0.219
Pseudocyst drainage	4 (19.0%)	9 (47.4%)	0.058
Pancreatic duct diameter (mm)	9.6 (4–15)	8.6 (5–20)	0.344
In-hospital stay (days)	14.4 (10–21)	18.9 (12–29)	0.001
Postoperative hospital stay (days)	8.1 (4–11)	11.1 (7–21)	0.002
Complications			
Postoperative pancreatic fistula	1 (4.8%)	2 (10.5%)	0.596
Surgical site infection	2 (9.5%)	3 (15.8%)	0.654
Delayed gastric emptying	1 (4.8%)	2(10.5%)	0.596
Upper gastrointestinal bleeding	1 (4.8%)	0(0%)	1.000
Reintervention rate	0	0	NA
Mortality rate %	0	0	NA
Insulin replacement requirement	0	0	NA
Exocrine pancreatic dysfunction	1 (4.8%)	1 (5.3%)	1.000
Pain relief rate %	100	100	NA

*NA* Not applicable

### The effect of pre-operative ESWL on robot-assisted Frey’s procedure

**Fig. 3:**
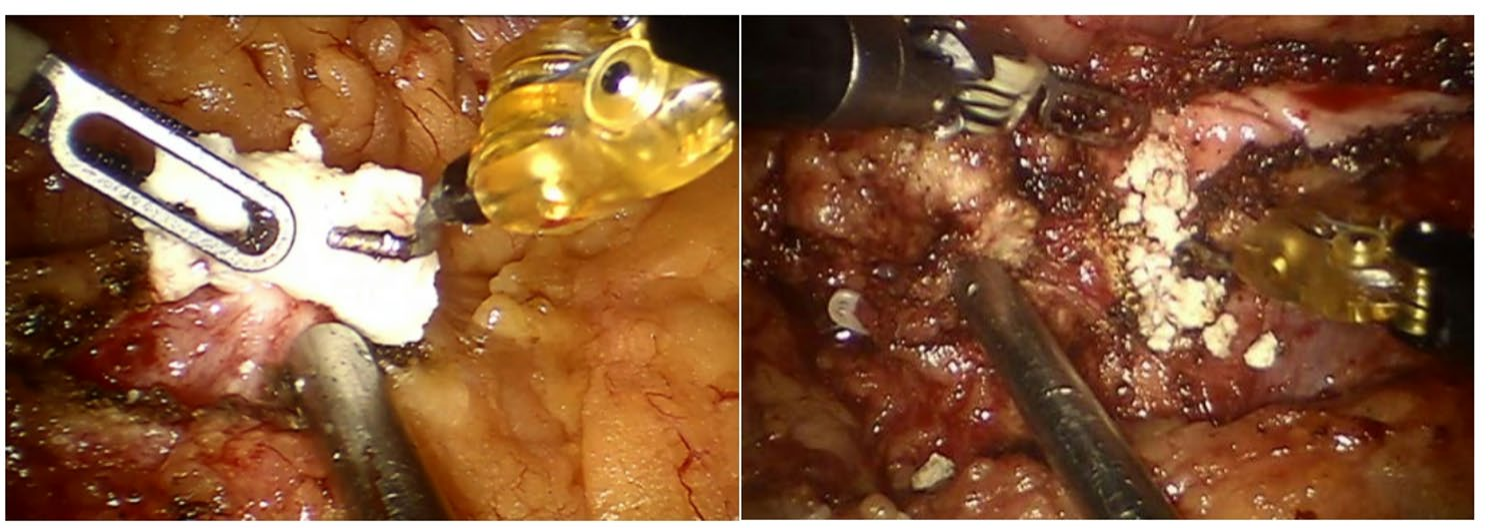
Pancreatic duct stones before and after ESWL

All in all, pre-operative ESWL had no significant effect on robot-assisted Frey’s procedure before and after surgery (Table 4). Figure 3 shows the images of pancreatic duct stones before and after ESWL. The operative time and postoperative hospital stay were almost the same whenever pre-operative ESWL was performed or not. There was an increasing trend of intraoperative bleeding after pre-operative ESWL (from 125 mL to 192 mL), but the difference was not statistically significant. After the pre-operative ESWL, the number of cases of pseudocyst drainage decreased from 4 to 0 (28.6% to 0, *P* > 0.05). There was no significant difference of the postoperative complications like postoperative pancreatic fistula, surgical site infection and delayed gastric emptying between the robot-assisted Frey’s procedure with and without pre-operative ESWL groups (*P* > 0.05). Meanwhile, the number of cases of upper gastrointestinal bleeding increased from 0 to 1. However, pre-operative ESWL will make it easier to remove the stone during surgery.

**Table 4:** The effect of pre-operative ESWL on the operative characteristics in robot-assisted Frey’s procedure group

Variable	Pre-operative ESWL (*n* = 7)	No pre-operative ESWL (*n* = 14)	*p* value
Operative time (min)	317 (180–540)	315 (240–420)	0.963
Intraoperative bleeding (mL)	192 (50–600)	125 (50–300)	0.251
Intraoperative transfusion	0	0	NA
Pseudocyst drainage	0 (0%)	4 (28.6%)	0.255
Pancreatic duct diameter (mm)	10.7 (8–15)	9.0 (4–15)	0.238
Postoperative hospital stay (days)	8.1 (4–11)	8.1 (4–10)	1.000
Complications			
Postoperative pancreatic fistula	0 (0%)	1 (7.1%)	1.000
Surgical Site infection	0 (0%)	2 (14.3%)	0.533
Delayed gastric emptying	0 (0%)	1 (7.1%)	1.000
Upper gastrointestinal bleeding	1 (14.3%)	0 (0%)	0.333

*ESWL* Extracorporeal shock wave lithotripsy, *NA* Not applicable

## Discussion

CP is an irreversible inflammatory process characterized by structural damage and fibrosis, eventually resulting in pancreatic exocrine and endocrine dysfunction. Frey’s surgery is one of the most performed procedures for chronic pancreatitis and can be performed open, laparoscopically or robotically [1]. Most of the previous literature reports were case reports, case series or reviews. In addition, data on robot-assisted Frey’s procedure is scarce. In this study, for the first time, we retrospectively reviewed the surgical information on CP of up to 40 patients who underwent Frey’s procedure (robotic and open) over a 7-year period at the First Affiliated Hospital of Xi’an Jiaotong University in China.

CP has a variety of etiologies and alcohol consumption is the leading cause worldwide (66%), followed by idiopathic, which reaches up to 29% [11]. However, our study offers a slightly different view. The results of this study showed that the most common cause was idiopathic, accounting for 47.5% (19 out of 40 patients) of all causes, followed by alcoholic etiology (32.5%). In fact, the incidence of idiopathic pancreatitis has increased concerning genetic etiology lately [12, 13]. The results of our study agree with those of others.

There are numerous special challenges associated with pancreatic surgery, mainly due to its proximity to vital blood vessels, the possibility of postoperative fistulas, and the fact that the pancreas is located in the retroperitoneum. Surgical approaches are mainly divided into resection, drainage and hybrid operation [3]. Hybrid procedures, including those performed by Beger’s and Frey’s, can be done by open, laparoscopic or robotic procedures [14]. Several randomized controlled trials have indicated that the Frey’s procedure is superior to other drainage and resection procedures for the treatment of CP in terms of long-term prognosis and associated morbidity [2]. In 2003, Giulianotti et al. were pioneers in publishing their experience with robotic pancreatoduodenectomy in Europe [15]; meanwhile, Melvin et al. described its application in pancreatic neuroendocrine tumors from the United States during that same year [16]. In recent years, robotic-assisted surgery has gradually been widely applied in pancreatic diseases because it is not only feasible, well-accepted and safe, but also shows an increasing trend in the treatment of pancreatic diseases and malignant tumors [8]. The results of our present study also confirmed that robotic surgery demonstrated superior characteristics compared to the Frey’s open surgery. The main advantages include less postoperative bleeding and shorter postoperative hospital stay.

ESWL was first used for the removal of renal calculi in 1980 [17] and the earliest description for its introduced in calcified chronic pancreatitis dates back to 1987 [18]. Some studied the efficacy of ESWL in the treatment of CP. In a meta-analysis by Moole et al., which included data of 27 studies of 3189 patients, reported a complete pain relief and an improved quality of life in 52,7% and 88,2% of pooled patients, respectively [19]. A recent meta-analysis by Van Huijgevoort et al. reported similar results, with a complete ductal clearance in 69,8% and a complete pain relief in 64,2% of pooled patients, respectively [20]. No conclusions were drawn about the effect of ESWL on exocrine and endocrine dysfunction, due to the heterogeneity of the different studies. Our results demonstrated that pre-operative ESWL does not increase the operation time and post-operative complications, but the stones are easier to remove. Meanwhile, our data illustrated that the case of postoperative upper gastrointestinal bleeding increased slightly in the pre-operative ESWL group, which may be due to inflammation.

In conclusion, CP is a progressive inflammatory disease characterized by structural damage and chronic pancreatic fibrosis, which is associated with pancreatic exocrine and endocrine dysfunction. Robotic Frey’s procedure is safe and feasible for CP patients. Preoperative ESWL treatment may be helpful for intraoperative pancreatic duct stone removal.

## Data Availability

The datasets used and/or analyzed during the current study are available from the corresponding author on reasonable request.

## Abbreviations

CP: Chronic pancreatitis
ESWL: Extracorporeal shock wave lithotripsy
SD: Standard deviation
ANOVA: One-way analysis of variance
